# Holmium:yttrium–aluminum–garnet laser–assisted retrieval of an embedded fully covered self-expandable metallic stent for benign biliary strictures

**DOI:** 10.1055/a-2893-7127

**Published:** 2026-07-08

**Authors:** Ippei Ikoma, Yusuke Takasaki, Haruka Hagiwara, Yasuhisa Jimbo, Sho Takahashi, Toshio Fujisawa, Hiroyuki Isayama

**Affiliations:** 1 Department of GastroenterologyGraduate School of Medicine, Juntendo UniversityTokyoJapan; 2 Division of GastroenterologyDivision of Gastroenterology, Department of Medicine, Faculty of MedicineBangkokThailand

## Acronyms and abbreviations



**Video 1** An embedded fully covered self-expandable metallic stent was successfully removed using peroral cholangioscopy-guided holmium:yttrium–aluminum–garnet laser ablation and lithotripsy.

BBS: Benign biliary strictures
FCSEMS: Fully covered self-expandable metallic stents
POCS: Peroral cholangioscopy
Ho:YAG: Holmium:yttrium-aluminum-garnet

Benign biliary strictures (BBSs) are commonly encountered after hepatobiliary surgery and require endoscopic stent placement. Fully covered self-expandable metallic stents (FCSEMSs) are increasingly used for this indication. 1​ However, FCSEMSs may become embedded in the bile duct wall, particularly after prolonged placement or disruption of the covering membrane. In such cases, stent removal can be extremely challenging.

The holmium:yttrium–aluminum–garnet (Ho:YAG) laser is effective for lithotripsy of pancreaticobiliary stones and tissue ablation. 2​ We report a case in which peroral cholangioscopy (POCS)-guided Ho:YAG laser ablation enabled the removal of an embedded FCSEMS.

**Fig. 1:**
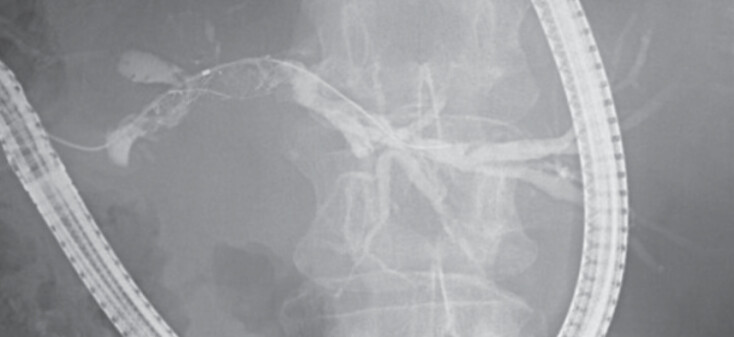
The fully covered self-expandable metallic stent (FCSEMS) caused focal biliary narrowing, with no passage of contrast medium.

A 48-year-old man underwent right hepatectomy for hepatocellular carcinoma and subsequently developed a postoperative BBS. An FCSEMS was placed to treat the BBS. Six months later, FCSEMS removal was attempted but was unsuccessful because of severe epithelial hyperplasia and partial stent embedding ( Fig. 1 ). Because repeated plastic stent exchange every 3–4 months was required to maintain biliary drainage, we decided to attempt the removal of the embedded FCSEMS using laser ablation ( Video 1 ).

**Fig. 2:**
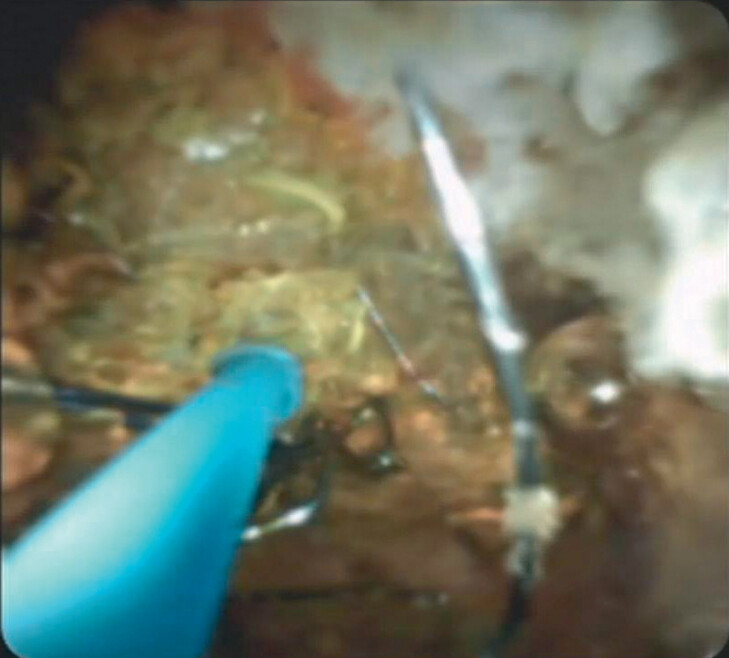
Peroral cholangioscopy-guided holmium:yttrium–aluminum–garnet laser lithotripsy was used to fragment the FCSEMS.

After biliary cannulation, a guidewire was advanced to the distal end of the stent. A POCS system (SpyGlass DS II; Boston Scientific, Marlborough, MA, USA) was inserted and revealed that epithelial hyperplasia had buried the stent. Ho:YAG laser ablation was then performed using a laser system (Litho EVO; EDAP TMS S.A., Vaulx-en-Velin, France) to dissect and ablate the ingrown hyperplastic tissue and expose the FCSEMS wires ( Fig. 2 ). The exposed metal mesh was fragmented using the laser and removed with forceps under direct POCS visualization.

**Fig. 3:**
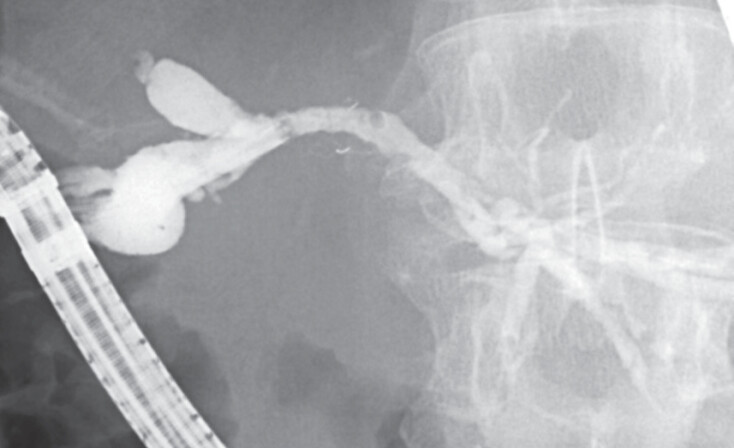
The FCSEMS was removed, and good contrast flow through the bile duct was observed.

The BBS was recanalized after complete stent removal, which was achieved over three sessions without procedure-related adverse events ( Fig. 3 ). This technique may represent an effective salvage option for recanalizing the BBS and retrieving the embedded FCSEMS.

Endoscopy_UCTN_Code_TTT_1AR_2AL
